# High-Energy Low-Velocity Impact Behavior of Rubber-Coated Sandwich Composite Structure with Buoyancy Material Core: Experimental and Numerical Investigation

**DOI:** 10.3390/ma18081791

**Published:** 2025-04-14

**Authors:** Yi Zhu, Zhiyuan Mei, Haitao Li, Hongbo Tao, Guotao Chen

**Affiliations:** 1 College of Naval Architecture and Ocean Engineering, Naval University of Engineering, Wuhan 430033, China; y_zhu96@163.com (Y.Z.);; 2 Luoyang Ship Material Research Institute, Luoyang 471023, China

**Keywords:** rubber-coated sandwich composite, buoyancy material core, impact response, failure mode, high-energy low-velocity impact

## Abstract

The dynamic response and failure of rubber-coated sandwich composite structures with buoyancy material core (RC-BMC-SCS) subjected to high-energy low-velocity impacts were experimentally and numerically investigated. Six types of BMC-SCSs were designed and manufactured, and high-energy low-velocity impact experiments were performed. Based on the Mohr-Coulomb theory and the Ogden hyperelasticity constitutive model, a low-velocity impact finite element analysis model was developed. The results indicate that BMC-SCS damage stages could be divided into: (1) matrix damage, (2) core cracks, (3) debonding and fiber breakage. Three distinct damage stages of the RC-BMC-SCS were revealed: (1) rubber layer energy absorption, (2) core cracks, (3) debonding. The rubber layer can enhance the damage threshold by approximately 100% compared to BMC-SCS. However, rubber energy absorption capacity has an upper limit. Additionally, the larger the curvature of the BMC-SCS, the higher the initial stiffness of the structure and the larger the impact damage area. The results of this study provide valuable insights for the multifunctional design of composite deep-sea marine structures.

## 1. Introduction

Sandwich composite structures (SCSs) are usually composed of fiber-reinforced plastic (FRP) upper and lower face sheets and lightweight core materials. Although this unique design reduces the overall quality of materials, it endows them with high bending stiffness. Moreover, the upper and lower face sheets play a protective and anti-corrosion role for the internal core materials, which is important for marine structures [1,2]. Meanwhile, buoyancy materials have been extensively developed and used in the field of deep-sea exploration due to their high strength, resistance to high hydrostatic pressure, and light weight [3]. SCSs with buoyancy material core (BMC-SCSs) have a wide range of applications in deep-sea structures, since they combine the advantages of both materials. However, with regard to assembly or maintenance operations in the complex and unknown deep-sea environment, collisions and other accidents inevitably occur. In 2007, the Mir submersible collided with a hydrothermal vent structure during a mid-Atlantic Ridge expedition due to operational error. The impact load exhibits typical high-energy and low-velocity characteristics, which can cause extensive internal damage, reduce the stiffness and strength of the composite structure [4,5,6], and potentially lead to catastrophic failure of the overall structure. In current low-velocity impact studies, the energy levels generally range from 10 to 100 Joules. It is still worth exploring whether high-energy low-velocity impacts, such as those more likely encountered by marine structures, can induce new damage modes and mechanisms.

To the best of the authors’ knowledge, and according to the available literature, the impact resistance of BMC-SCSs has been rarely reported. SCSs are commonly used with core materials such as polyurethane (PU) foam, polymethacrylimide (PMI) foam, and various structural grid forms. For instance, Long et al. [7] conducted systematic experimental and numerical studies on the impact resistance of PU foam/glass fiber-reinforced polymer (GFRP) sandwich structures with different impact energies, foam densities, and layup sequences. It was found that the delamination failure of the upper face sheet follows the behavior typical of composite laminates, while penetration changes the shape of the delamination region. Compared to those with soft cores, sandwich structures with rigid cores are more prone to delamination. Chen et al. [8] developed a PU foam/GFRP grid sandwich structure, and their experimental results revealed that the damage modes of the sandwich beams were multiple and strongly related to the impact position. Reducing the transverse and longitudinal web spacing and increasing the web thickness can enhance the impact resistance of sandwich structures, with the boundary conditions having a significant effect on the post-damage stage. Lv et al. [9] performed drop hammer low-velocity impact tests on PMI foam/CFRP grid sandwich structures at different locations, e.g., intersection, web, and center, using different energy levels. The intersection location exhibits the best impact resistance. Morada et al. [10] investigated sandwich structures consisting of alumina tri-hydrate (ATH)/epoxy core and GFRP panels. Their results indicated that the core material was the main energy absorbing part of the structure. In addition, the peak impact force was approximately proportional to the impact energy. Due to their unique configuration, the impact resistance of grid sandwich structures is closely related to the impact location [11,12,13,14,15]. He et al. [16] conducted experimental and simulation studies on an X-Frame aluminum alloy core/CFRP sandwich structure. In this configuration, under low impact energy at a small-span location, the upper face sheet around the impact area primarily exhibited interlayer delamination. As the impact energy increased, intra-layer damage, e.g., fiber fracture and matrix cracking, became the dominant damage mode. Li et al. [17] studied a Kagome grid core material sandwich structure and conducted drop hammer experiments with three types of impactors. They found that the shape of the impactor had a significant effect on the damage patterns of the sandwich panels, with the flat impactor causing more severe damage.

The new BMC used in this study is a synthetic material, which is generally made of a resin matrix filled with hollow glass microspheres; changes in the resin matrix properties and hollow glass microsphere content can produce BMCs with different characteristics [18,19,20]. The buoyancy material failure criterion must be analyzed based on its specific composition and experimental results. Li et al. [21] studied the compression characteristics of an epoxy-based buoyancy material and identified two main coexisting damage modes; one was the fracture of the dominant hollow glass microspheres at the central part, and the other was the formation and propagation of shear cracks from the corners. Furthermore, the increase in the strength of the epoxy matrix with strain rate accounted for the macroscopic strain-rate dependence of the buoyancy material. Zhang et al. [22] conducted similar work on the same system and developed a corresponding finite element (FE) analysis model based on experimental data. Based on uniaxial tensile/compression experimental results, Zhai et al. [23] established a double Drucker-Prager failure criterion for epoxy-based buoyancy materials considering tensile/compression differences. Gupta et al. [24] compared the tensile/compression properties of vinyl ester-based buoyancy materials with those of epoxy-based buoyancy materials. They reported that low-cost vinyl ester resins can produce buoyancy materials with properties comparable to those of epoxy-based systems.

Given that various types of cracking damage occurred in the buoyancy blocks used in the Jiaolong deep-sea manned submersible during the 35th and 37th China Oceanic Research Expeditions [25], rubber or polyurea can be used as impact-resistant protective layers for further protection of the BMC-SCS [26,27,28,29,30]. Therefore, this paper proposes a rubber-coated (RC) BMC-SCS (RC-BMC-SCS) for deep-sea marine structures and investigates its impact resistance characteristics. The impact process is captured by force sensors, laser displacement sensors, and high-speed cameras. The damage morphology of the sandwich structure after impact is analyzed via waterjet cutting or computerized tomography (CT) technology. The effects of curvature and impact energy on the impact resistance of the sandwich structure are discussed, and the protection mechanism of the rubber is analyzed. Subsequently, a low-velocity impact FE model of a typical specimen is developed based on the experimental results, revealing the damage mechanism of the sandwich structure. Using this model, further efforts are made to explore the relationship between impact resistance and the impact energy or thickness of the rubber.

## 2. Experimental Methodology

### 2.1. Manufacturing Process

**Figure 1:**
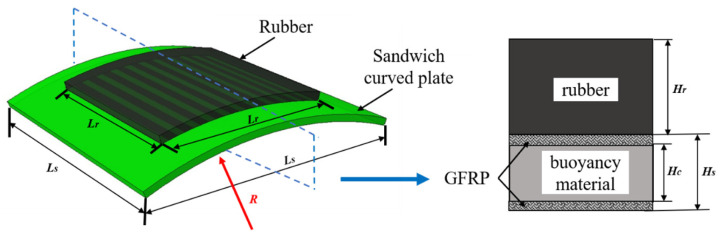
Schematic illustration of the RC-BMC-SCS specimen.

The RC-BMC-SCS was prepared as a single curved plate made of a BMC sandwich plate with a rubber sheet bonded on it (Figure 1). The curvature radius of the BMC sandwich plate was *R*, its length and width span were *L*_s_, its total thickness was *H_s_*, its core thickness was *H_c_*, and the thickness of the upper and lower face sheets (GFRP) was the same. To ensure proper fastening between the sandwich structure and the low-velocity impact test tooling, the rubber sheet did not cover the entire sandwich plate; its length and width were *L_r_*, and its thickness was *H_r_*. The details of the different specimens are listed in Table 1, where R0/R12-1/R12-2/R6-1/R6-2 are the control samples. In the standard ASTM D7136 [31], the inplane dimensions of the test specimen were 150 mm × 100 mm, and the diameter of the impactor was 16 mm. In this study, to reflect the impact characteristics of marine structures, the diameter of the hemispherical impactor was 80 mm. Correspondingly, to reduce the influence of the boundaries, the specimen size *L_s_* was enlarged to 450 mm.

**Figure 2:**
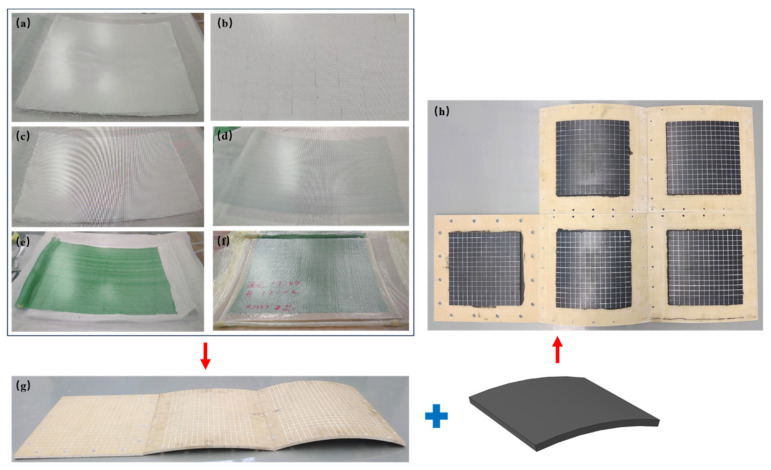
Fabrication of the RC-BMC-SCS. (**a**) Laying the upper face sheet fiberglass fabric; (**b**) laying the buoyancy material block; (**c**) laying the lower face sheet fiberglass fabric; (**d**) laying the release cloth; (**e**) setting the deflector mesh; (**f**) bag mold sealing and resin infusion; (**g**) BMC-SCS; (**h**) RC-BMC-SCS.

The BMC-SCS panel was fabricated using GFRP upper and lower face sheets and the BMC material via vacuum-assisted resin infusion in a single curing process. Before infusion curing, a wooden negative mold with proper curvature and size was produced. The process involved sequentially laying the upper face sheet fiberglass fabric, buoyancy material block, and the lower face sheet fiberglass fabric, followed by the application of the release cloth, deflector mesh, bag mold sealing, and finally resin infusion (Figure 2a–f). Among them, the fabric was S2 high-strength fiberglass satin fabric (Sinoma Technology Co., Ltd., Suzhou, China) with an areal density of 220 g/m^2^, [0/90] orthogonal layup, and a single-layer thickness of about 0.21 mm. The buoyancy material was a hollow glass microspheres/epoxy resin composite (Shanghai Qianjin New Material Technology Co., Ltd., Shanghai, China) with a density of 700 kg/m^3^. The resin matrix during the curing process was 430LV vinyl resin (Jinling Liliansi Resin Co., Ltd., Nanjing, China). After curing and molding, nitrile rubber (System Engineering Research Institute of China Shipbuilding Group Corporation, Beijing, China) was bonded to the upper face sheet of the BMC sandwich plate (Figure 2g,h).

### 2.2. High-Energy Low-Velocity Impact Tests

**Figure 3:**
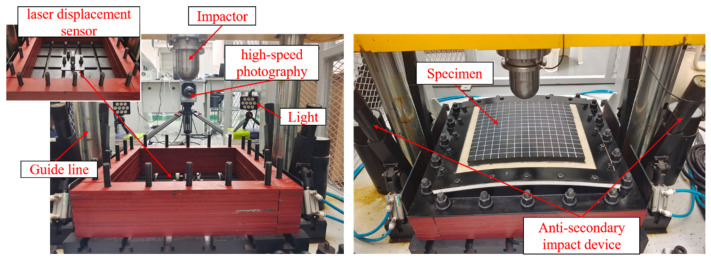
Low-velocity test set-up.

In the high-energy low-velocity impact experiments, an STM STLS-10000 drop hammer test machine with an anti-secondary impact device was used. The hemispherical impactor mass was increased or decreased through counterweights with a mass range of 100–200 kg. The impact velocity was controlled by adjusting the height of the impactor; the maximum drop height was 5 m. A DHDAS dynamic signal acquisition and analysis system collected force–time and displacement–time data. The force sensor was RIGHT T401, and the acquired signals were amplified through a charge amplifier (RIGHT T499). The laser displacement sensor was DPTEX CD33. To record the entire impact process in detail, a Photron FASTCAM SA-Z high-speed camera was utilized to capture the deformation of the specimens under impact. The experimental platform and specimen tooling are depicted in Figure 3. During impact, the specimen was fastened by a steel pressure plate and screws. The different experimental cases are listed in Table 1. In the tests, the drop heights H were 0.2 m and 0.45 m, corresponding to drop velocities of 2 m/s and 3 m/s.

### 2.3. Damage Inspection

A combination of destructive and non-destructive tests (NDTs) was used to assess impact damage. The NDTs included visual and industrial CT inspection. Ultrasonic phased array detection was not used since the thin sandwich structure panels cause significant interference from surface-reflected acoustic waves, while the buoyancy materials and rubber absorb the high-frequency acoustic beams (center frequency of 5 MHz). Considering the subsequent residual strength characterization, industrial CT was used to detect the damage in the R0, R12-2, R0-R, and R12-2-R specimens, while the remaining specimens were subjected to destructive testing to observe cross-sectional damage patterns.

#### 2.3.1. Visual Inspection

**Figure 4:**
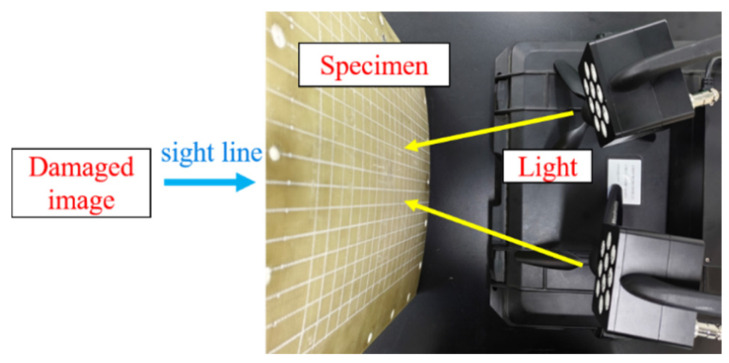
Visual inspection of damaged area via strong light irradiation.

Visual inspection of impact and rear surface damage is the most straightforward method, primarily focusing on identifying morphological changes. Given the good light transmittance of the GFRP face sheets, a strong light was used to irradiate the impact surface of the specimen with no rubber layer, enabling the observation of the damage characteristics and area from the back side (Figure 4).

#### 2.3.2. CT Inspection

**Figure 5:**
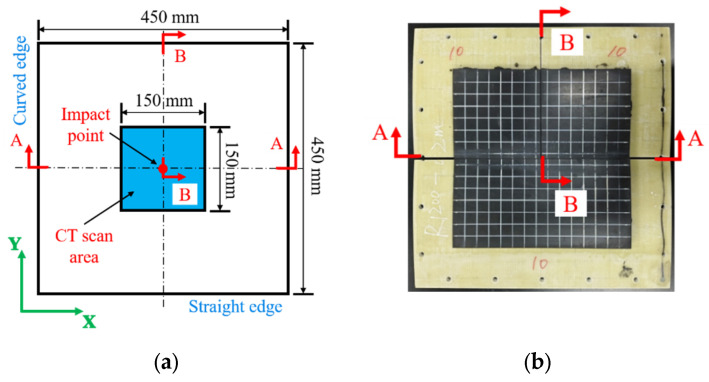
Schematic diagrams of CT scanning and waterjet cutting. (**a**) CT scanning; (**b**) waterjet cutting. The green coordinate system represents the CT scanning coordinate system. The Z-axis is perpendicular to the paper surface, following the right-hand rule.

To avoid destruction of the specimen structure, an industrial-grade NanoVoxel CT device (Sanying Precision Instruments Co., Ltd., Tianjin, China) was utilized to observe the internal damage patterns after impact (Figure 5a). To improve the resolution (71 μm), the swept area was narrowed down to the center area of the specimen.

#### 2.3.3. Destructive Testing

As shown in Figure 5b, after the NDT was completed, waterjet cutting was used to cut the impact specimens along the A-A and B-B sections, enabling the visual observation of the cross-sectional damage morphologies.

## 3. Experimental Results and Discussion

### 3.1. Failure Mode

The damage mechanisms were systematically categorized into three distinct modes: face sheet damage, core crack, and interfacial debonding. Specifically, the face sheet damage mechanism was further classified into two primary failure modes: matrix-dominated damage (manifested as surface whitening through resin microcracking) and fiber fracture (characterized by visible through-thickness cracks). A critical differentiation was made between core cracking (initiating within the core material) and interfacial debonding (propagating along the face sheet-core interface) based on fracture propagation characteristics.

**Figure 6:**
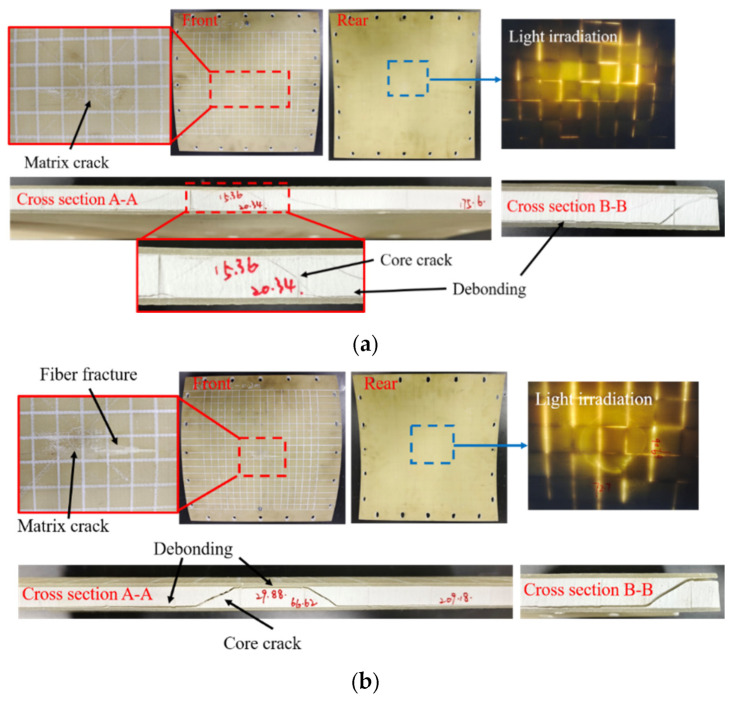

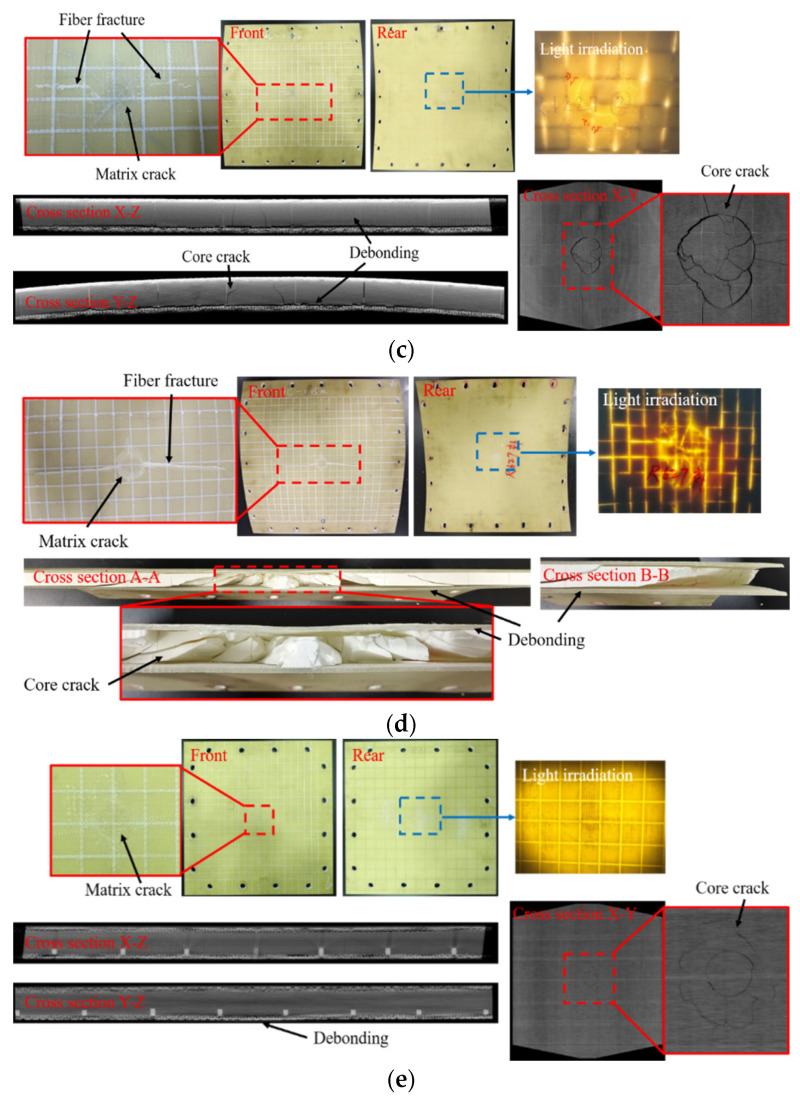
Damage patterns of BMC-SCSs impacted at different energy levels. (**a**) R12-1; (**b**) R6-1; (**c**) R12-2; (**d**) R6-2; (**e**) R0.

Figure 6 exhibits the damage patterns of the sandwich structure without a rubber layer under low-velocity impact at different energy levels. It can be observed that the damage of the sandwich structure increased with increasing impact velocity. Under the impact condition of 100 kg^−2^ m/s, the R12-1 specimen exhibited no apparent surface damage, and the upper and lower face sheets were intact. Only the matrix of the upper face sheet in the impact area was damaged due to the localized contact effect, while no obvious core damage was visible in the light image. As shown in Figure 6a, the waterjet-cut profile revealed that four symmetric cracks were generated in the core due to shear stress, which extended to the interface between the core and the lower face sheet, causing debonding damage. The core damage in R6-1 was more severe than that in R12-1 under the same impact conditions, and cracks were visible in the light image (Figure 6b). In addition to matrix damage in the upper face sheet, fiber breakage occurred, and significant shear cracks appeared in the core, leading to debonding at the adhesive interface between the upper face sheet and the core near the impact point. On the other hand, no debonding occurred between the lower face sheet and the core in the impact region. The visual damage characteristics of R12-2 and R6-2 (impact case 100 kg^−3^ m/s) were almost the same as those of R12-1 and R6-1. The main differences were that the matrix damage and indentation at the impact position were more obvious, the fiber breakage area was wider, the core damage range observed in the light image was larger, and the damage was more severe. As depicted in Figure 6c,d, the obvious core damage range contour in R6-2 was not clearly observable. The CT image of R12-2 revealed a roughly circular core damage contour, with radial longitudinal cracks appearing at the periphery. Complete debonding between the lower face sheet and the core was observed within the CT view. The core damage in R6-2 exhibited fragmentation and complete debonding of the upper and lower face sheets but still retained the shear damage characteristics. R0, impacted at a velocity of 3 m/s, exhibited a more pronounced two-ringed circular outer contour in the core damage revealed in the CT image, with fewer outwardly radiating cracks compared to R12-2.

**Figure 7:**
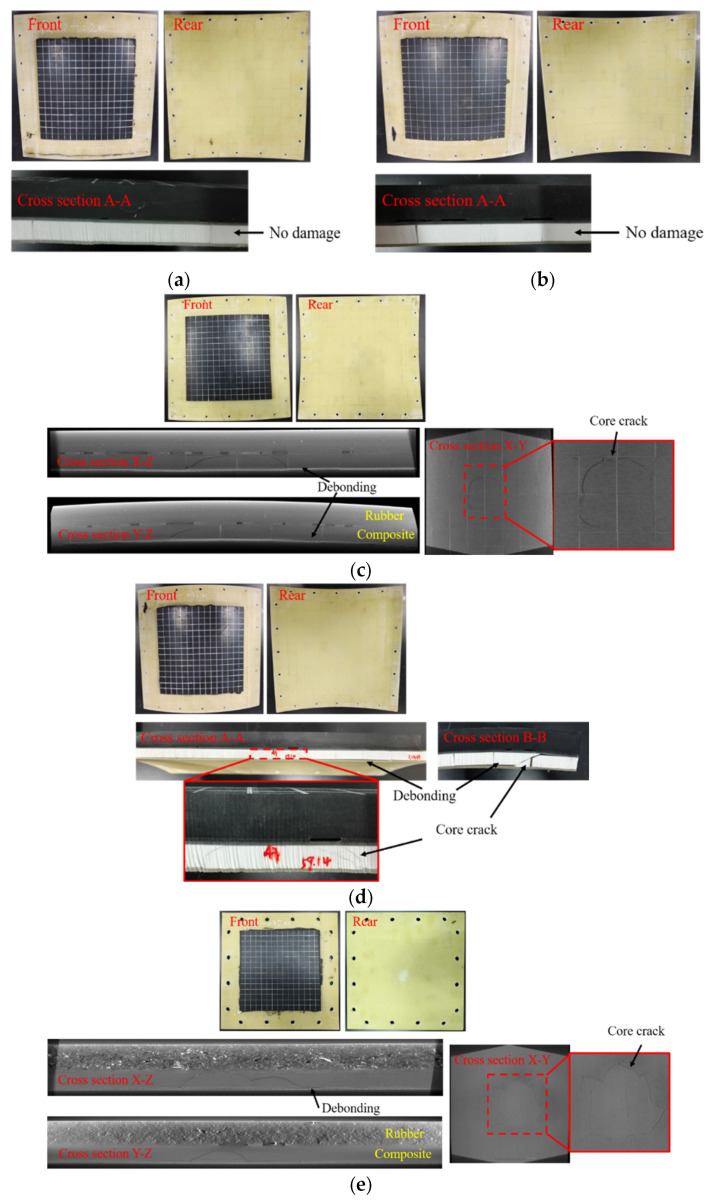
Damage patterns of the RC-BMC-SCSs impacted at different energy levels. (**a**) R12-1-R; (**b**) R6-1-R; (**c**) R12-2-R; (**d**) R6-2-R; (**e**) R0-R.

Figure 7 demonstrates the damage patterns of RC-BMC-SCS specimens subjected to high-energy low-velocity impact. The R12-1-R and R6-1-R specimens exhibited no apparent damage under an impact velocity of 2 m/s (Figure 7a,b). When the impact velocity was increased to 3 m/s, there was still no visible surface damage on the R12-2-R, R6-2-R, and R0-R specimens. According to the CT images (Figure 7c,e), shear damage occurred in the cores of R12-2-R and R0-R, with a damage morphology similar to that of R12-1. This damage was accompanied by debonding between the core and both the upper and lower face sheets. The damage of R6-2-R was slightly more severe than that of R12-2-R, with pronounced cracking in the core.

**Figure 8:**
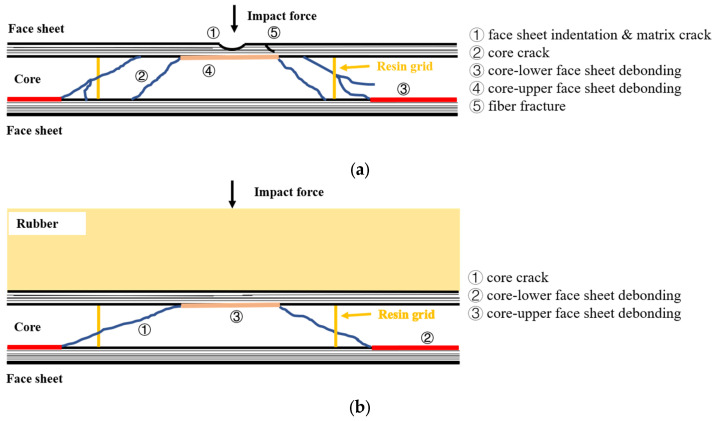
Typical damage patterns of BMC-SCS. (**a**) BMC-SCS; (**b**) RC-BMC-SCS.

Figure 8a summarizes the above BMC-SCS damage modes, including indentation and matrix damage, core crack, which may turn along the resin grid, upper face sheet/core debonding, core/lower face sheet debonding, and fiber fracture. Figure 8b summarizes the BMC-SCS damage modes, including core crack, upper face sheet/core debonding, and core/lower face sheet debonding. The measured data of each damage mode are listed in Table 2. Comparative analysis revealed that, under high-energy low-velocity impacts, the rubber layer can, within a certain range, effectively absorb energy and enhance impact resistance, improving the damage threshold while preventing contact damage to the upper face sheet. With the increase in the impact energy, the outer contour of the core crack shifts gradually from distinct to crushed cracks, and the debonding area at the interface between the lower face sheet and the core expands. Moreover, it can be found that the debonding area between the upper face sheet and the core increases significantly with increasing curvature.

### 3.2. Impact Response

#### 3.2.1. Impact Response of Sandwich Structure Without Rubber Layer

**Figure 9:**
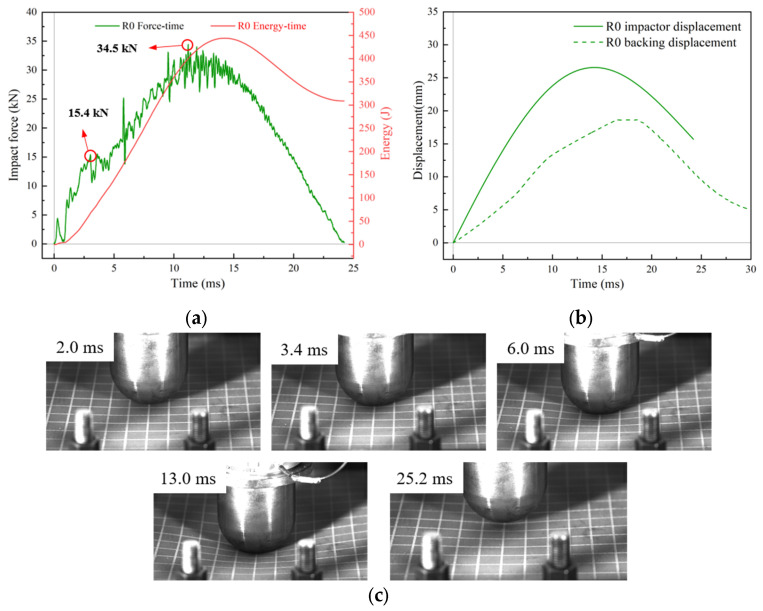
Low-velocity impact response of flat sandwich plate without rubber layer. (**a**) Impact force/energy vs. time curves; (**b**) Displacement vs. time curves; (**c**) Deformation process of R0.

Figure 9 shows the response of R0 after impact at a velocity of 3 m/s. The impact force–time curves and displacement–time curves were both convex, indicating that no penetration occurred (Figure 9a,b). At the onset of impact, the sandwich plate was deformed as a whole, and the impact area was concave. At this point, the upper face sheet matrix was damaged due to the contact effect. The impact force increased rapidly with time, and at t = 3.4 ms, the impact force reached 15.4 kN. Under the action of membrane stress and bending stress, the core was damaged, elastic energy was released, and the upper face sheet, except for the impact point, rebounded (Figure 9c). At the same time, the local stiffness decreased, and the impact force decreased rapidly. As the impact process progressed, local and global deformation occurred simultaneously. Furthermore, fluctuations appeared with increasing impact force, indicating that damage continued to progress. This damage mainly comprised two modes: debonding between the lower face sheet and the core after initial damage, and shear failure of the undamaged portion of the core material. At t = 13 ms, the impact force reached its maximum value of 34.5 kN, the impact velocity dropped to 0, and the impactor began to move in the opposite direction. Clear indentations and plastic deformation were observed in the sandwich panel when the impactor detached from the BMC-SCS. The energy–time curve in Figure 9a reflects the energy absorption and release process of the sandwich structure subjected to the low-velocity impact. Figure 9b exhibits the impactor displacement–time curve, i.e., the deformation history of the impacted surface, and the backing plate center displacement–time curve. It can be seen that the displacement of the impactor was uniformly larger than that of the backing plate center, indicating that the thickness of the structure decreased during the impact process, as expected. The peak shift in the backing plate center displacement–time curve and the lack of smoothness in the curve were due to the low sampling frequency in the initial experiments, which was adjusted accordingly in the subsequent tests.

**Figure 10:**
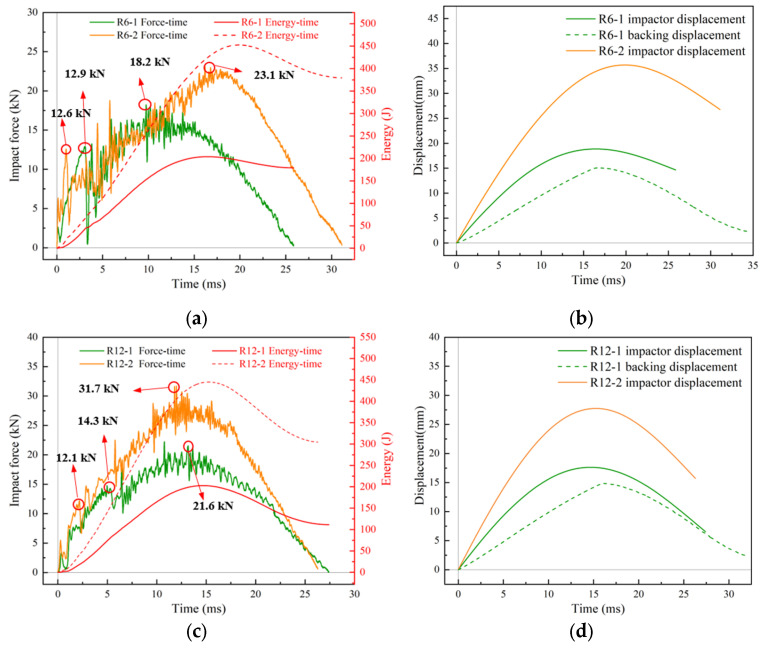

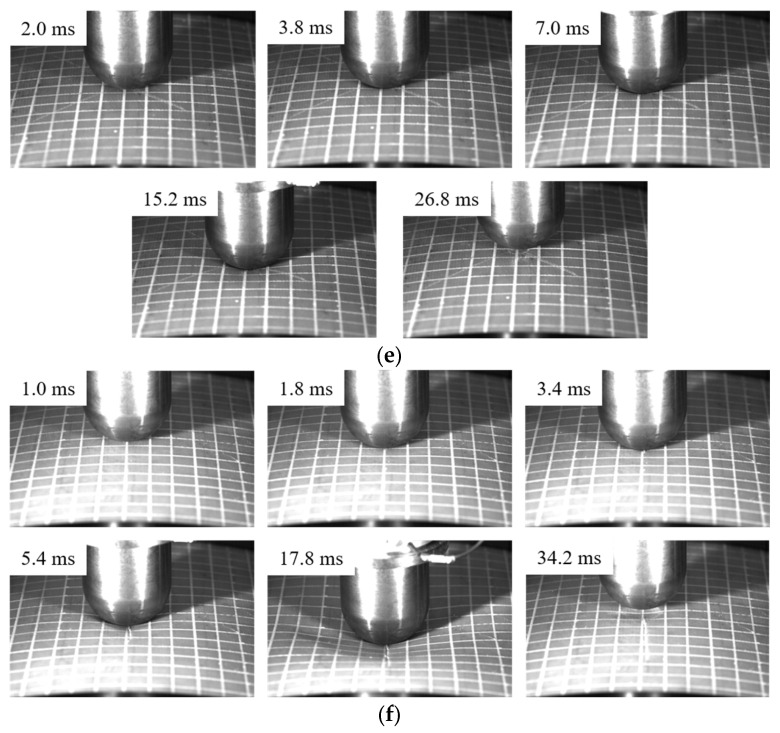
Low-velocity impact response of curved sandwich plate without rubber layer. (**a**,**c**) Impact force/energy-time curves; (**b**,**d**) displacement–time curves; deformation process of (**e**) R6-1 and (**f**) R6-2.

Figure 10 demonstrates the responses of R6-1/R12-1 and R6-2/R12-2 after impact at 100 kg^−2^ m/s and 100 kg^−3^ m/s, respectively. The characteristics of the impact force/energy–time curves and the displacement–time curves were similar to those of R0. In particular, the duration of the impact became longer, and the maximum displacement of the impactor increased with increasing impact velocity. The impact force–time curve exhibited a slight fluctuation before reaching the damage threshold, which was due to structural vibration and other factors. At the damage threshold, the impact force decreased sharply, followed by a strengthening process. Subsequently, the curve rose gradually with large fluctuations until reaching the maximum value, after which it began to decline (Figure 10a–d). The peak force of the initial damage of the core of R6-1 occurred at t = 3.8 ms, while that of R6-2 occurred earlier at t = 1.8 ms. The two peaks were close to each other, i.e., 12.9 kN and 12.6 kN, respectively. The initial damage peaks of R12-1 and R12-2 were slightly different, i.e., 14.3 kN (t = 5.6 ms) and 12.1 kN (t = 3 ms). The maximum peak of the impact force of R12-2 (31.7 kN) was significantly higher than that of R6-2 (23.1 kN), whereas that of R12-1 (21.6 kN) was closer to that of R6-1 (18.2 kN), which may be related to both impact energy and curvature, as discussed in Section 3.3 and Section 3.4. Figure 10e,f depict the high-speed camera images of the impact process of R6-1 and R6-2. In both images, an overall rebound phenomenon of the upper face sheet was observed after the initial damage to the core. In addition, the increase in curvature caused a more pronounced localized impact deformation in the sandwich panels compared to the high-speed camera images of R0. In Figure 10f, the fiber fracture process of the R6-2 upper face sheet can be observed. At t = 3.4 ms, fiber fracture arose at the outer edge of the impact indentation. Due to the effects of the boundary conditions, the structural strain was larger along the straight edge, causing the crack to gradually expand along this direction. The maximum deflection was reached at t = 17.8 ms, when the length of the crack also reached its maximum. At this point, the peak difference between the displacement–time curves of the R6-1 backing plate center and the impactor was 3.8 mm, while that of R12-1 was 2.7 mm. This indirectly indicates that the structure was damaged at this point, with an indentation in the upper face sheet.

#### 3.2.2. Impact Response of Sandwich Structures with Rubber Layer

**Figure 11:**
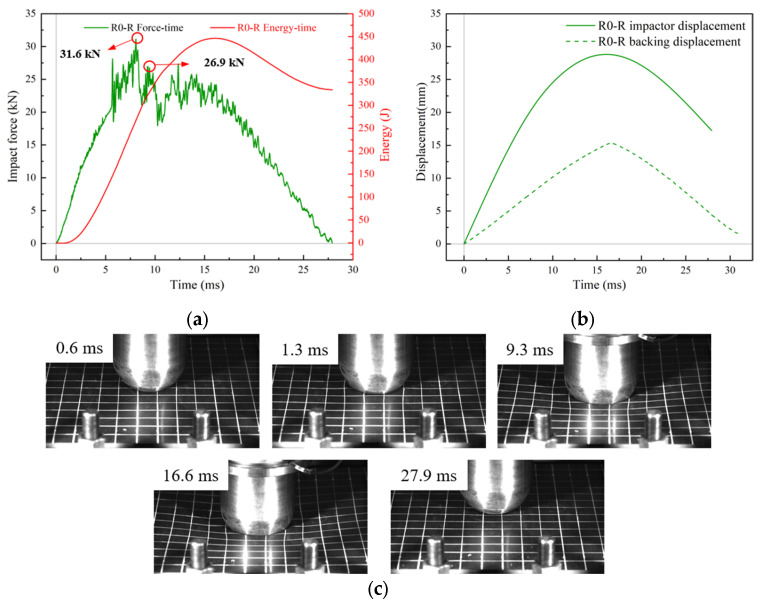
Low-velocity impact response of flat RC-BMC-SCS plate. (**a**) Impact force/energy–time curves; (**b**) displacement–time curves; (**c**) deformation process of R0-R.

By comparing the impact force-time curves in Figure 9a and Figure 11a, an obvious difference between the dynamic responses of R0-R and R0 can be found. Due to the presence of the rubber layer, the fluctuations and signal burrs of the impact force–time curve were reduced, as the rubber played a filtering role. The overall shape of the curve changed from convex to multi-peak. The impact force continued to increase after the initial impact, with a sudden drop occurring only at the maximum peak. Analysis of the damage pattern revealed that, under the effects of debonding between the core and the lower face sheet, the impact force exhibited multiple peaks, all smaller than the initial peak. After reaching the maximum deformation, the curve began to decline as the impactor started to move in the reverse direction. As depicted in Figure 11c, at the beginning of impact, the rubber layer was in contact with the impactor; thus, only the rubber layer was deformed at this point, while the plate underwent no apparent deformation. As the impact progressed, the sandwich plate and the rubber layer were deformed as a whole, while the relative motion between the rubber and the impactor continued to exist. At about t = 7.6 ms, the impact force peaked at 31.6 kN, followed by several less intense peaks, which indicated that the sustained damage process had begun. The high-speed images revealed the release of elastic energy and the rebound process of the rubber and the upper face sheet at t = 9.3 ms. At t = 16.6 ms, the displacement of the impactor reached its maximum value. At the end of the impact process, no apparent indentations or areas of damage were found on the rubber surface. The peak difference between the displacement–time curves of the backing plate and the impactor reached 13.5 mm (Figure 11b). This is attributed to the fact that the modulus of the impactor was much higher than that of the rubber, making the rubber prone to undergo large deformation upon contact.

**Figure 12:**
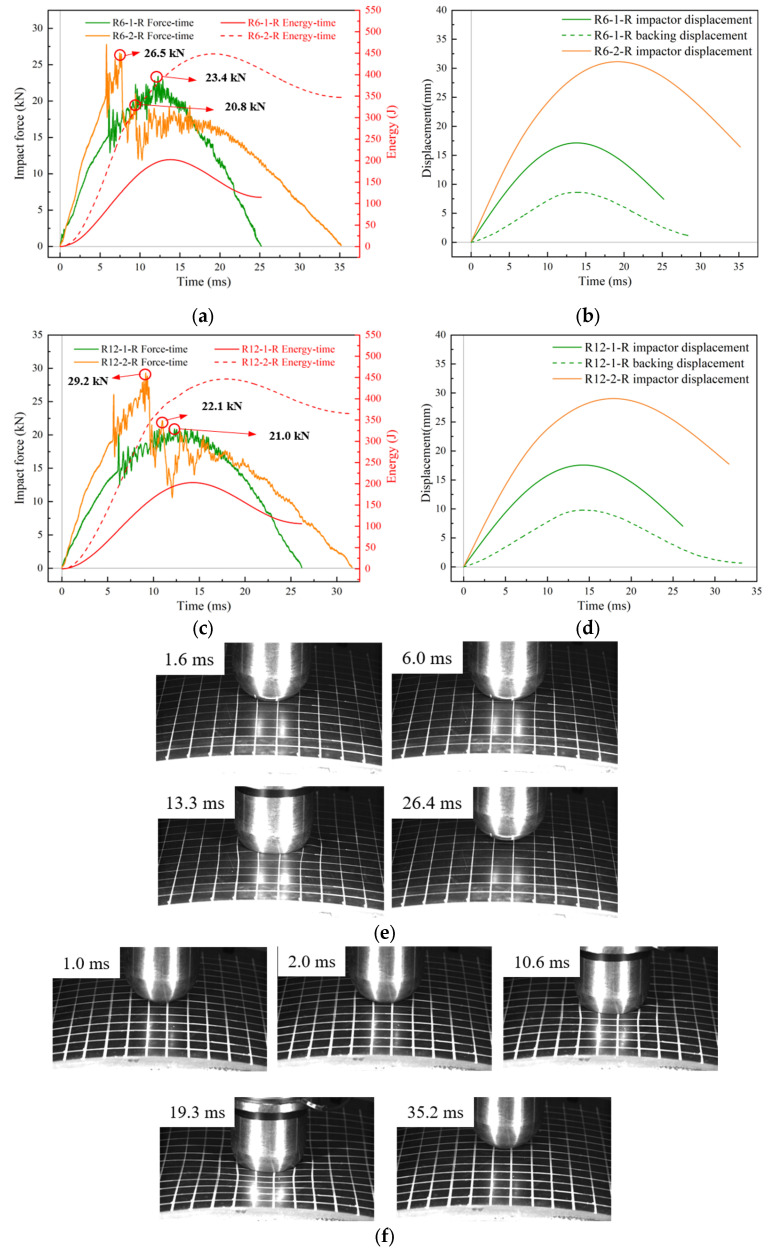
Low-velocity impact response of curved RC-BMC-SCS plate. (**a**,**c**) Impact force/energy–time curves; (**b**,**d**) displacement–time curves; deformation process of (**e**) R6-1-R and (**f**) R6-2-R.

The impact force–time curves of the R6-1-R and R12-1-R specimens in Figure 12a,c exhibited clear elastic response characteristics, symmetric about the maximum peak axis, while the impact force response of the R6-2-R and R12-2-R specimens was similar to that of R0-R. According to the high-speed camera images for R6-1-R and R6-2-R (Figure 12e,f), at t = 10.6 ms, R6-2-R exhibited overall rebound and release of the upper face sheet and the rubber layer, while this phenomenon was not observed in R6-1-R. When the impact velocity was increased, the impact duration, maximum impactor displacement, and maximum impact force increased (Figure 12a–d). The maximum impact forces of R6-1-R and R12-1-R were 23.4 kN and 22.1 kN, respectively. In addition, the maximum impact force of R6-2-R (26.5 kN) was slightly lower than that of R12-2-R (29.2 kN). According to Figure 12b,d, the peak difference between the displacement–time curves of the backing plate and the impactor for R6-1-R was 8.5 mm, and that for R12-1-R was 7.8 mm. These values were much larger than those of R6-1 and R12-1, which is consistent with the findings for R0-R.

### 3.3. Effects of Curvature

**Figure 13:**
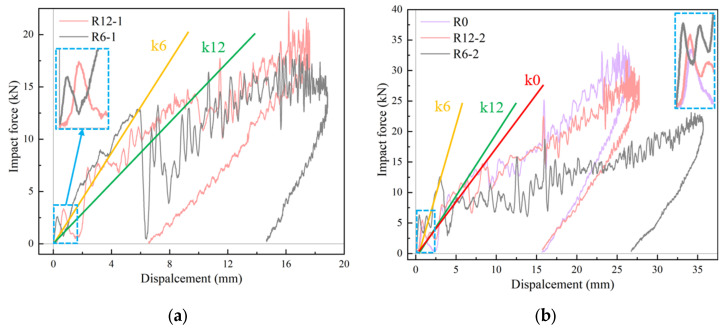

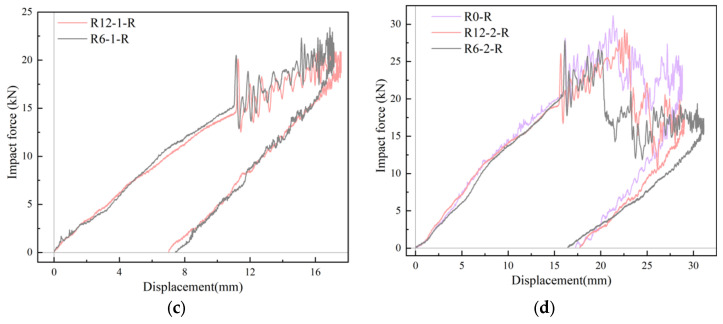
Effects of curvature on impact force–displacement curves. (**a**) Impact velocity of 2 m/s without rubber; (**b**) impact velocity of 3 m/s without rubber; (**c**) impact velocity of 2 m/s with rubber; (**d**) impact velocity of 3 m/s with rubber.

Figure 13 depicts the impact force–displacement curves at different impact velocities, reflecting the stiffness change and energy absorption characteristics of the BMC-SCS. According to Figure 13a,b, the initial load was reduced to 0 after 2.5 kN, indicating that the 1.5 mm thick GFRP plate was damaged. However, the local face sheet damage had little effect on the overall stiffness of the structure. The core crack is defined as the initial damage that has a significant impact on the overall stiffness and damage evolution. The initial stiffness is the stiffness before the occurrence of the initial damage, which followed the order R6-1 > R12-1 and R6-2 > R12-2 > R0 and was clearly affected by the curvature. The initial damage occurred earlier in R6, with a smaller initial damage displacement and less energy absorbed at this stage. After the occurrence of local damage and stiffness degradation, the damage expanded outward, forming a wider overall damage area; thus, the impact displacement in R6 was the largest, and the damage was more severe.

No damage occurred in R12-1-R and R6-1-R. In the ideal case, the impact force–displacement curve in that region should be 0. Nevertheless, in Figure 13c, energy absorption can be observed, which can be attributed to one of the following three reasons: first, the displacement values on the horizontal axis are derived from the integral of the impact force curve, which may contain high-frequency noise and other errors; second, the bolts used to fix the boundary may have caused energy dissipation; third, the sandwich structure may have undergone slight plastic deformation, absorbing part of the energy. In Figure 13c,d, it can be observed that the initial stiffness of the system composed of the curved plate and the rubber layer was dominated by the rubber, which had a significantly lower modulus; therefore, at this point, the curve was smooth. When the impact force increased, after the rubber reached its energy absorption threshold, the stiffness of the sandwich plate became the dominant factor. At this point, the effect of curvature was reflected in the curve, and a burr signal appeared in the curve. Hence, in Figure 13d, R6-2-R experienced the initial damage, and the impactor reached the maximum displacement due to severe stiffness degradation. On the contrary, in Figure 13c, the impact–displacement curves nearly overlapped since no damage occurred, with only minor differences in the main region of action of the plate.

### 3.4. Effects of Rubber Coating

**Figure 14:**
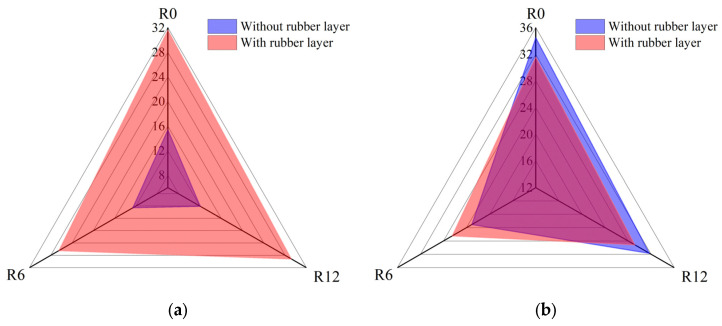
Effects of rubber layer on impact force. (**a**) Initial damage impact force threshold; (**b**) peak impact force (unit: kN).

Figure 14 demonstrates the effects of the rubber layer on the impact forces. As depicted in Figure 14a, the apparent initial damage impact force threshold of the RC-BMC-SCS significantly increased. This was because the rubber layer itself could deform to absorb the impact energy, playing a protective role by reducing the “point contact” and stress concentration between the impactor and the upper face sheet. This is also the reason why the debonding area between the upper face sheet and the core of the RC-BMC-SCS increased. In Figure 14b, the peak impact force of the sandwich structure without the rubber layer was larger due to the emergence of sustained damage and the strengthening process after initial damage. With regard to the RC-BMC-SCS, due to stiffness degradation, the rubber continued to deform and absorb energy, the debonding between the lower face sheet and the core dissipated energy, and there was no core sustained damage phenomenon; thus, the maximum value was the initial damage impact force threshold.

### 3.5. Energy Absorption

**Figure 15:**
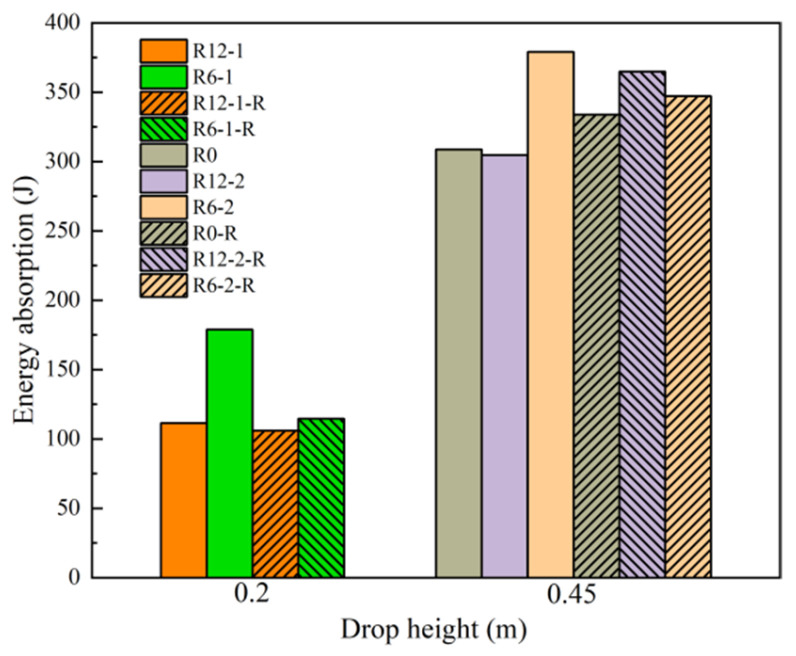
Energy absorption of the sandwich structure in each case.

The energy absorption levels of the BMC-SCSs with and without a rubber layer are presented in Figure 15. It can be clearly observed that the energy absorption level of the sandwich structure increased with increasing impact velocity, leading to more severe damage. In the cases without a rubber layer, the larger the curvature, the higher the energy absorption. For example, R6-1 exhibited 61.3% higher energy absorption than R12-1, while R0 and R12-2 presented similar levels. Moreover, R6-2 absorbed 24.7% more energy than R12-2. On the other hand, in the presence of a rubber layer, the curvature had little effect on energy absorption.

## 4. FE Modeling and Analysis

### 4.1. Constitutive Model

A three-dimensional strain-based failure criterion for woven fabric composites [32] was employed to simulate the different face sheet failure modes, including fiber fracture, crack damage, and delamination. The specific damage criteria and material parameters are given in Table 3 and Table 4, respectively. After reaching the failure criterion, the panel did not fail immediately but maintained its bearing capacity, with its mechanical properties gradually deteriorating until complete failure. The damage criteria and progressive degradation of laminates were implemented into a user-defined material subroutine (VUMAT).

**Table 3:** Material model damage initiation criteria.

Tension-shear fiber modes	$f_{7}-{r_{7}}^{2}={\left(\frac{E_{a}\langle \varepsilon_{a}\rangle }{S_{aT}}\right)}^{2}+{\left(\frac{G_{ac}\langle \varepsilon_{ac}\rangle }{S_{aFS}}\right)}^{2}-{r_{7}}^{2}=0$	(1)
	$f_{8}-{r_{8}}^{2}={\left(\frac{E_{b}\langle \varepsilon_{b}\rangle }{S_{bT}}\right)}^{2}+{\left(\frac{G_{bc}\langle \varepsilon_{bc}\rangle }{S_{bFS}}\right)}^{2}-{r_{8}}^{2}=0$ $S_{aFS}=S_{FS},S_{bFS}=S_{FS}\times S_{bT}/S_{aT}$	(2)
Compression fiber modes	$f_{9}-{r_{9}}^{2}={\left(\frac{E_{a}\langle \varepsilon_{a}^{\prime }\rangle }{S_{aC}}\right)}^{2}-{r_{9}}^{2}=0,\;\varepsilon_{a}^{\prime }=-\varepsilon_{a}-\langle -\varepsilon_{c}\rangle \frac{E_{c}}{E_{a}}$	(3)
	$f_{10}-{r_{10}}^{2}={\left(\frac{E_{b}\langle \varepsilon_{b}^{\prime }\rangle }{S_{bC}}\right)}^{2}-{r_{10}}^{2}=0,\;\varepsilon_{b}^{\prime }=-\varepsilon_{b}-\langle -\varepsilon_{c}\rangle \frac{E_{c}}{E_{b}}$	(4)
Crush mode	$f_{11}-{r_{11}}^{2}={\left(\frac{E_{c}\langle -\varepsilon_{c}\rangle }{S_{FC}}\right)}^{2}-{r_{11}}^{2}=0$	(5)
In-plane matrix mode	$f_{12}-{r_{12}}^{2}={\left(\frac{G_{ab}\varepsilon_{ab}}{S_{ab}}\right)}^{2}-{r_{12}}^{2}=0$	(6)
Delaminations	$\begin{array}{l} f_{13}-{r_{13}}^{2}=\\ S^{2}\times \left\{{\left(\frac{E_{c}\langle \varepsilon_{c}\rangle }{S_{cT}}\right)}^{2}+{\left(\frac{G_{bc}\varepsilon_{bc}}{S_{bc0}+S_{SRC}}\right)}^{2}+{\left(\frac{G_{ac}\varepsilon_{ac}}{S_{ac0}+S_{SRC}}\right)}^{2}\right\}-{r_{13}}^{2}=0 \end{array}$	(7)

Note: ⟨⟩ are Macaulay brackets.

**Table 4:** Material model input parameters for glass woven composite.

Symbol	Property	Value
ρ (kg/m^3^)	Density	1750
$E_{a}$, $E_{b}$ (GPa)	Elastic modulus 1	22
$E_{c}$ (GPa)	Elastic modulus 3	8
$\nu_{ab}$	Poisson’s ratio 12	0.13
$\nu_{bc}$, $\nu_{ac}$	Poisson’s ratio 13	0.2
$G_{ab}$ (GPa)	Shear modulus 12	4
$G_{ac}$, $G_{bc}$ (GPa)	Shear modulus 13	6
$S_{aT}$ (MPa)	Tensile strength 1	550
$S_{aC}$ (MPa)	Compressive strength 1	332
$S_{bT}$ (MPa)	Tensile strength 2	550
$S_{bC}$ (MPa)	Compressive strength 2	332
$S_{cT}$ (MPa)	Through thickness tensile strength	58 [33]
$S_{FC}$ (MPa)	Crush strength	850 [33]
$S_{FS}$ (MPa)	Fiber mode shear strength	300 [33]
$S_{ab}$ (MPa)	Matrix mode shear strength plane 12	102 [33]
$S_{ac}$, $S_{bc}$ (MPa)	Matrix mode shear strength planes 23 and 31	58 [33]
S	Delamination criterion scale factor	1.2 [33]

The debonding failure between the face sheet and the core was described by the quadratic failure criterion and the B-K law [34] to predict the initiation and propagation of debonding, as expressed by Equations (8) and (9), respectively.

(8) $${\left\{\frac{\langle \tau_{n}\rangle }{N}\right\}}^{2}+{\left\{\frac{\tau_{s}}{S}\right\}}^{2}+{\left\{\frac{\langle \tau_{t}\rangle }{T}\right\}}^{2}=1,$$

Here, $\tau_{n}$ and *N* represent the normal traction and strength, $\tau_{s}$ and $\tau_{t}$, and *S* and *T* represent the shear tractions and shear strengths.

(9) $$G_{C}=G_{IC}+\left(G_{IIC}-G_{IC}\right){\left\{\frac{G_{shear}}{G_{T}}\right\}}^{\eta },$$

Here, $G_{shear}=G_{IIC}+G_{IIIC}$, $G_{T}=G_{shear}+G_{IC}$, and $G_{IC}$ is the normal critical fracture energy, $G_{IIC}$ and $G_{IIIC}$ are the shear critical fracture energy, respectively, and *η* is the relevant coefficient in the B-K law. The specific material parameters are listed in Table 5.

**Figure 16:**
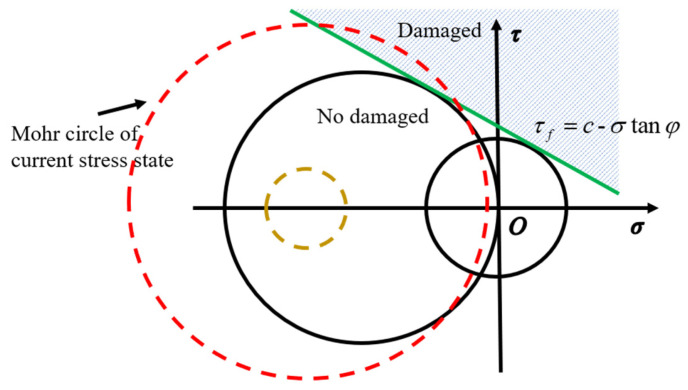
Mohr-Coulomb theory.

The buoyancy material used in this study exhibited brittle crack characteristics, with cracks occurring at an angle to the uniaxial compressive loading direction. Therefore, core damage was characterized using the Mohr-Coulomb theory, which considers both compressive and shear stresses [35]:

(10) $$\tau_{f}=c-\sigma \tan\varphi ,$$

where c is the cohesive force and φ is the internal friction angle. As illustrated in Figure 16, a straight line (green line) can be obtained by fitting pure shear to the uniaxial compressive stress state. If the Mohr circle intersects with this straight line, it is considered that the core material has reached the failure condition, and damage occurs. The required material parameters are shown in Table 6. The corresponding damage criterion was also implemented into VUMAT.

Rubber is a hyperelastic material. Its large deformation during the impact process can be simulated by the 3rd-order Ogden constitutive model [36] to avoid convergence problems.

(11) $$W=\sum_{i=1}^{N}\frac{\mu_{i}}{\alpha_{i}}\left(\lambda_{1}^{\alpha_{i}}+\lambda_{2}^{\alpha_{i}}+\lambda_{3}^{\alpha_{i}}-3\right),$$

**Figure 17:**
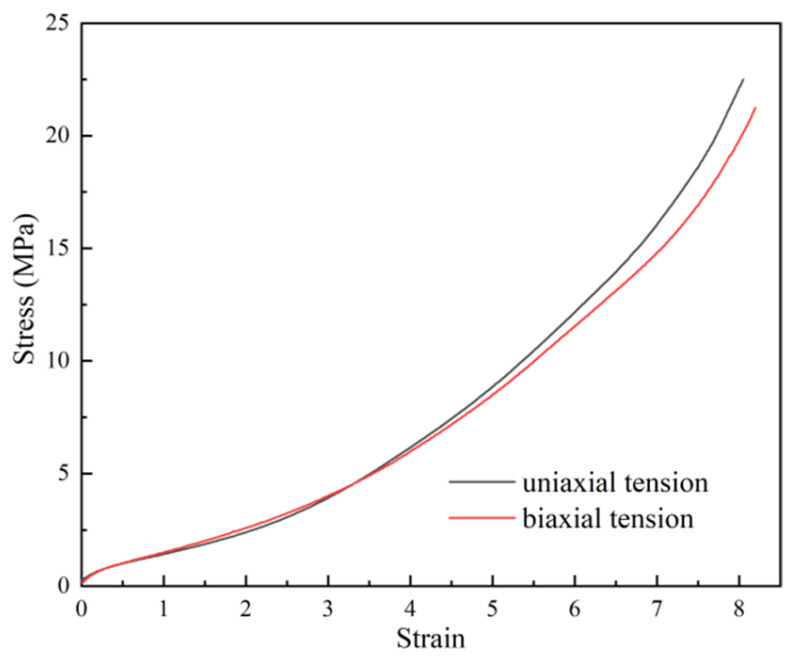
Uniaxial/biaxial tensile stress-strain curves of rubber.

Here, $\mu_{i}$ and $\alpha_{i}$ are the parameters. The uniaxial and biaxial tensile testing results of the rubber are presented in Figure 17. The specific parameters obtained through fitting are given in Table 7.

### 4.2. Modelling Details

**Figure 18:**
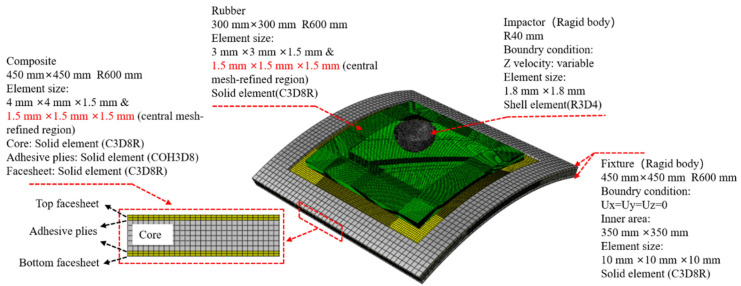
Schematic illustration of the FE model.

**Figure 19:**
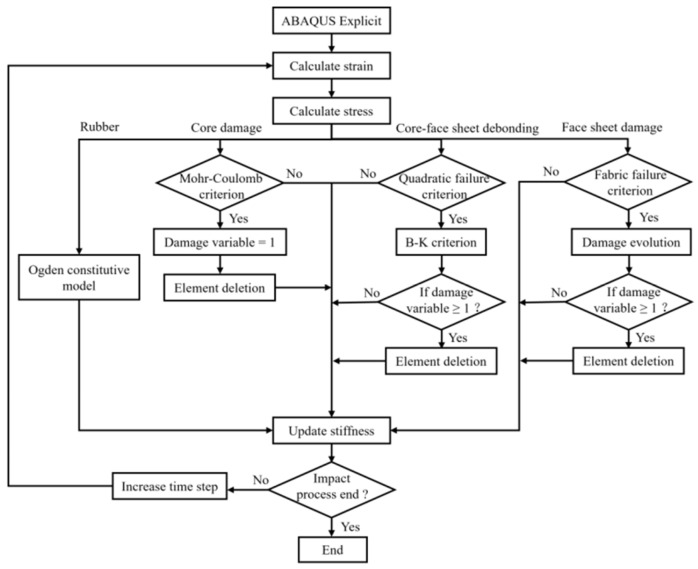
Flowchart of the simulation process.

The ABAQUS 2022/Explicit FE analysis software was used to numerically simulate the high-energy low-velocity impact response of a typical specimen (R6-R). Figure 18 illustrates the model in detail, including dimensions, material properties, and boundary conditions. Reduced integral elements (C3D8R) were used to mesh the GFRP, buoyancy material, and rubber. Stiffness control was applied to the elements to reduce the hourglass effect. Cohesive elements (COH3D8) were used to mesh the adhesive plies. The impactor was set as a rigid body and meshed with discrete rigid body elements (R3D4). According to the mesh convergence analysis, considering the accuracy and efficiency of the calculation, a fine mesh with elements measuring 1.5 mm × 1.5 mm × 1.5 mm was used near the impact area, while a coarser mesh was used in the other parts. The simulation process is presented in Figure 19.

### 4.3. FEM Validation

**Figure 20:**
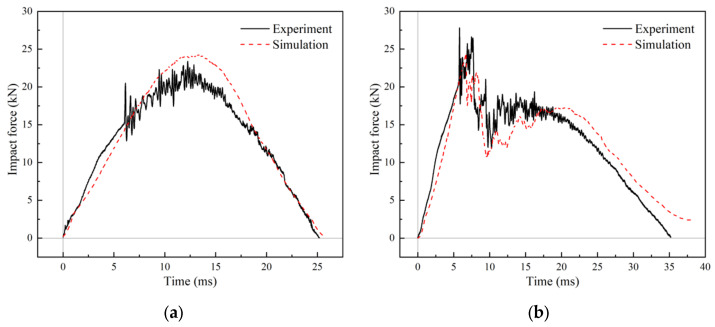
Comparison between experimental and simulation results. (**a**) R6-1-R; (**b**) R6-2-R.

**Figure 21:**
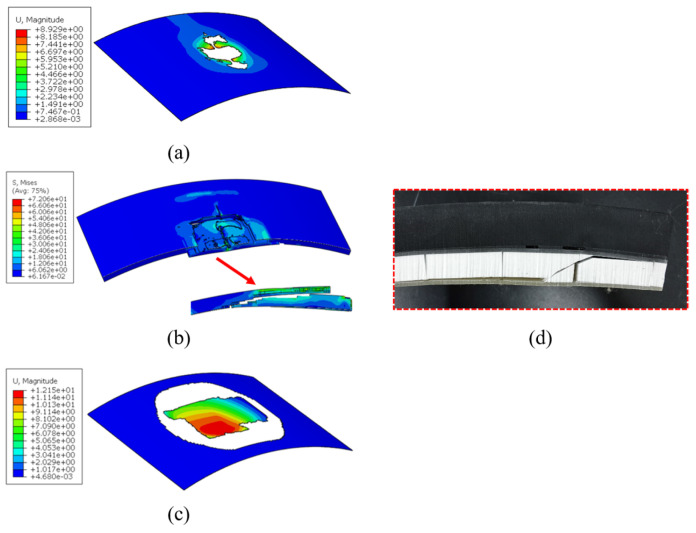
Comparison of the damage zones observed experimentally and predicted numerically for the R6-2-R specimen. (**a**) Upper adhesive ply debonding; (**b**) lower adhesive ply debonding; (**c**) core fracture; (**d**) R6-2-R damage pattern.

Figure 20 compares the experimental and FE simulation results of the impact response of the R6-1-R and R6-2-R specimens. The FE simulation impact force–time curve agreed well with the experimental one. Figure 21 illustrates the impact damage morphological characteristics of R6-2-R obtained via FE simulation. The FE model accurately reflected the debonding characteristics between the upper face sheet and the core, as well as those between the core and the lower face sheet, the absence of debonding in the impact area, and the shear damage on the core surface. The FE model ignored the effects of BMC chunking, exhibiting a more idealized result. Manufacturing defects, the fine structure of the composite material, and other factors may have caused deviations in the predictions.

### 4.4. Parametric Analysis

After a collision occurs, technicians need to quickly assess the damage. At this point, the initial impact damage threshold is particularly important for determining whether damage has occurred. To systematically investigate the impact resistance of RC-BMC-SCS, the effects of impact energy and rubber layer thickness under different conditions were assessed using the R6-R model.

#### 4.4.1. Effects of Impact Energy

**Figure 22:**
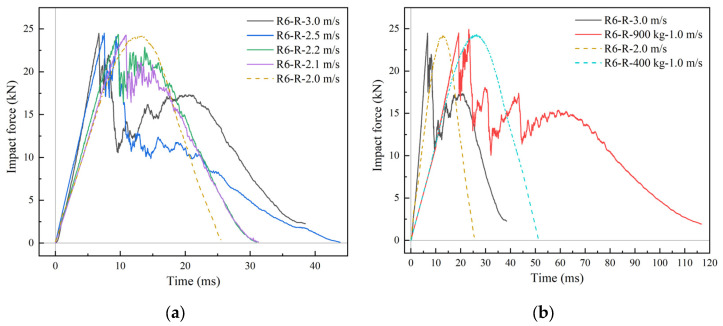
Effect of impact energy on impact force. (**a**) Different impact energy levels; (**b**) different impact impulse levels.

According to the R6-2-R simulation results (Figure 22a) for different impact velocities and energies, the maximum impact force in all cases was about 24 kN. The structural form and initial stiffness of the BMC-SCS were the same, while the damage was dominated by the core material. The stiffness of the structure degraded sharply after damage, with the maximum impact force occurring at the moment of initial damage. Therefore, all cases exhibited similar maximum values, while the impact force–time curve of 100 kg^−2^ m/s demonstrated elastic characteristics, indicating that the critical impact velocity was between 2 m/s and 2.1 m/s. In Figure 22b, the impact energy was kept constant, while the impact velocity and impactor mass were adjusted to change the impact impulse. It was found that using the impact energy as a criterion for determining whether damage will occur is more intuitive. The impulse of R6-R-400 kg-1.0 m/s was higher than that of R6-R-3.0 m/s, while the impact energy of R6-R-3.0 m/s was higher. It is obvious that the damage occurs in R6-R-3.0 m/s, and the initial damage threshold of R6-R impact was about 200 J. In addition, when the impact energy was the same, the peak value of the impact force remained almost unchanged, and the effect of impulse was mainly reflected in the impact time. In general, the larger the impulse, the longer the impact duration. In elasticity, the duration is approximately proportional to the impulse. For example, the impulse of the R6-R-400 kg-1.0 m/s case was twice that of the R6-R-2.0 m/s case, and the duration increased from 25 ms to 50 ms.

#### 4.4.2. Effects of Rubber Layer Thickness

**Figure 23:**
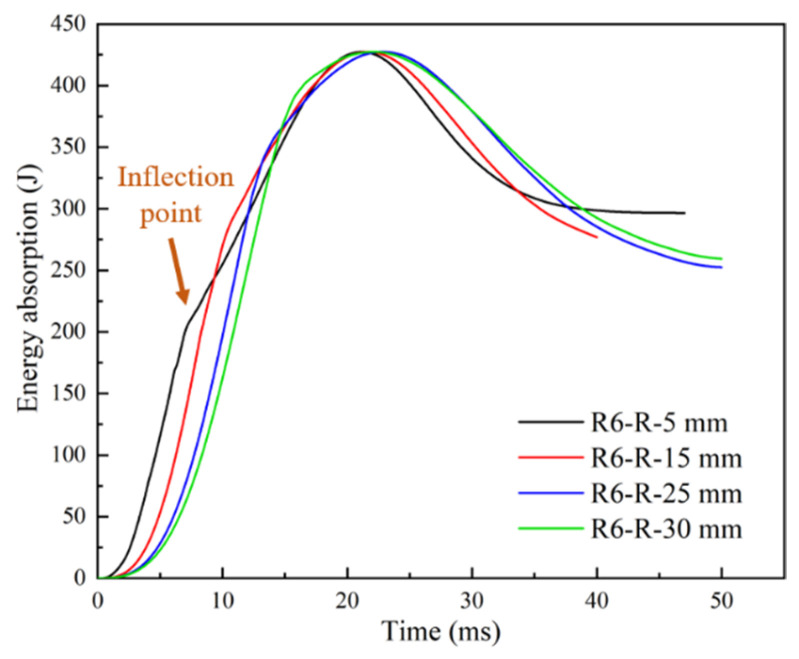
Effects of rubber layer thickness on energy absorption.

**Figure 24:**
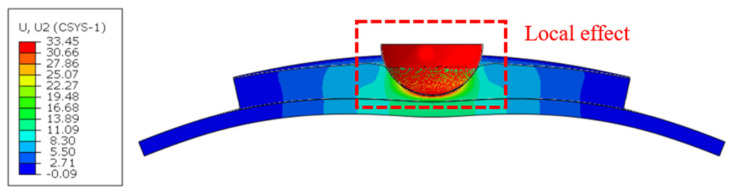
Rubber layer vertical deformation during impact in the R6-R-25 mm case (unit: mm).

Figure 23 presents the relationship between energy absorption and rubber layer thickness. The curves exhibited obvious inflection points, indicating that, at this point, the core material underwent brittle fracture. The maximum energy absorption values of all curves were similar. At this point, the velocity of the impactor dropped to 0. The maximum energy absorption value did not reach 450 J due to the presence of hourglass control, which introduced artificial pseudo strain energy in the calculation process. As the rubber layer thickness increased, the energy absorption time of the rubber increased, causing the curve to shift significantly backward. Although damage of the sandwich structure still occurred, indicating that the rubber layer thickness could be increased to a certain extent to improve impact performance, it could not prevent the occurrence of damage in high-energy low-velocity impacts. Furthermore, there was a gradual increase in energy absorption with increasing rubber layer thickness, but this increase reached a saturation point. This is due to the fact that the rubber only produced a local energy-absorbing effect (Figure 24), which was relatively limited compared to the total energy of the impact. For example, in the R6-R-25 mm case, the rubber absorbed a maximum energy of 142.3 J during the impact, accounting for 31.6% of the total energy. In high-energy low-velocity impact scenarios, where the mass of the impactor is much larger than that of the structure, the sandwich plate remains the main load-bearing and energy storage (consumption) structure.

## 5. Conclusions

In this paper, the impact response and failure mechanisms of RC-BMC-SCSs subjected to high-energy low-velocity impacts were investigated. Based on experimental observations and analyses, a VUMAT subroutine based on the fabric lamina damage criterion and Mohr–Coulomb theory was employed to model the damage behavior of the structure. The impact force–time curves and BMC damage patterns obtained via simulation agree well with the experimental results. The main conclusions are as follows:

(1) Impact resistance of RC-BMC-SCSs is mainly determined by the BMC. The crack initiation from the impact damage occurs in the core and gradually expands throughout the impact process, leading to debonding between the upper and lower face sheets and the core.
(2) The damage of BMC-SCSs initiates with indentation and matrix, which is mainly attributed to the contact effect. Subsequent core cracks, face sheet/core debonding, and fiber breakage occur.
(3) The rubber layer can significantly enhance the damage threshold by approximately 100%. The rubber layer provides local buffering, prevents contact damage to the upper face sheet, and expands the contact area between the impactor and the structure. Based on the results of the present research, it is recommended to use impact energy as the initial damage criterion for high-energy low-velocity impacts of RC-BMC-SCSs. However, rubber energy absorption capacity has an upper limit and does not increase indefinitely with thickness.
(4) The larger the curvature of the BMC-SCS, the higher the initial stiffness of the sandwich structure, and the earlier the occurrence of initial damage and stiffness degradation. The rubber layer can eliminate the effects of curvature to a certain extent.

## Figures and Tables

**Table 1:** Details of low-velocity impact specimens with different characteristics.

No.	*L_s_* (mm)	*H_s_* (mm)	*H_c_* (mm)	*R* (mm)	*L_r_* (mm)	*H_r_* (mm)	*H* (m)	*M* (kg)	Impact Energy (J)
R0	450	12	9	∞	300	-	0.45	100	450
R12-1	450	12	9	1200	300	-	0.20		200
R12-2	450	12	9	1200	300	-	0.45		450
R6-1	450	12	9	600	300	-	0.20		200
R6-2	450	12	9	600	300	-	0.45		450
R0-R	450	12	9	∞	300	15	0.45		450
R12-1-R	450	12	9	1200	300	15	0.20		200
R12-2-R	450	12	9	1200	300	15	0.45		450
R6-1-R	450	12	9	600	300	15	0.20		200
R6-2-R	450	12	9	600	300	15	0.45		450

Note: The “∞” in the *R* column indicates a flat plate, the “-” in the *H_r_* column indicates no rubber, and “*H*” and “*M*” denote the drop height and mass of the impactor, respectively.

**Table 2:** Quantification of typical damage patterns (Figure 8) of different specimens.

No.	①	②	③	④	⑤
R12-1	√	clear-cut outline	175.6–20.3	15.4	×
R6-1	√	clear-cut outline	209.2–66.6	29.9	√
R12-2	√	comminuted crack	OR–0	24.7	√
R6-2	√	comminuted crack	365.9–0	176.7	√
R0	√	comminuted crack	OR–26.2	25.4	×
R12-1-R	-	-	-	-	-
R6-1-R	-	-	-	-	-
R12-2-R	×	clear-cut outline	OR–60.2	25.3	×
R6-2-R	×	clear-cut outline	348.5–59.1	43.0	×
R0-R	×	clear-cut outline	OR–65.0	22.4	×

Note: The above units are all in mm. In column ① and ⑤, “√” and “×” indicate yes/no to the presence of this damage pattern, respectively. In column ③, “X–Y” indicates the total range of debonding between the lower face sheet and the core (X) and the undebonded portion at the impact area (Y). “OR” indicates that the value is beyond the range of the CT scanning area.

**Table 5:** Material parameters for debonding failure.

Symbol	Property	Value
*N* (MPa)	Interlaminar strength	30
*S*, *T* (MPa)		40
$G_{IC}$ (N/mm)	Critical fracture toughness	0.43
$G_{IIC}$$,\;G_{IIIC}$ (N/mm)		1.52
*η*	B-K coefficient	1.45

**Table 6:** Material parameters for core crack.

Symbol	Property	Value
$\rho_{core}$ (kg/m^3^)	Density	700
$E_{core}$ (GPa)	Modulus	2.5
$\nu_{core}$	Poisson’s ratio	0.31
$S_{coreS}$ (MPa)	Shear strength	34.5
$S_{coreC}$ (MPa)	Compressive strength	104.9

**Table 7:** Material parameters for Ogden model.

Symbol	Value
$\mu_{1}$ (MPa)	0.72387
$\mu_{2}$ (MPa)	0.09776
$\mu_{3}$ (MPa)	−0.000084
$\alpha_{1}$	1.4747
$\alpha_{2}$	3.6771
$\alpha_{3}$	−2.9281

## Data Availability

The original contributions presented in this study are included in the article. Further inquiries can be directed to the corresponding author.
